# Systems for deep brain stimulation: review of technical features

**DOI:** 10.1007/s00702-017-1751-6

**Published:** 2017-07-13

**Authors:** A. Amon, F. Alesch

**Affiliations:** Medical University of Vienna, Vienna, Austria

**Keywords:** Deep brain stimulation, Movement disorders, Psychiatric disorders, Technical features, Programming, Hardware

## Abstract

The use of deep brain stimulation (DBS) is an important treatment option for movement disorders and other medical conditions. Today, three major manufacturers provide implantable systems for DBS. Although the underlying principle is basically the same for all available systems, the differences in the technical features vary considerably. This article outlines aspects regarding the technical features of DBS systems. The differences between voltage and current sources are addressed and their effect on stimulation is shown. To maintain clinical benefit and minimize side effects the stimulation field has to be adapted to the requirements of the patient. Shaping of the stimulation field can be achieved by the electrode design and polarity configuration. Furthermore, the electric signal consisting of stimulation rate, stimulation amplitude and pulse width affect the stimulation field. Interleaving stimulation is an additional concept, which permits improved treatment outcomes. Therefore, the electrode design, the polarity, the electric signal, and the concept of interleaving stimulation are presented. The investigated systems can be also categorized as rechargeable and non-rechargeable, which is briefly discussed. Options for interconnecting different system components from various manufacturers are presented. The present paper summarizes the technical features and their combination possibilities, which can have a major impact on the therapeutic effect.

## Introduction

Since its introduction more than 25 years ago (Benabid et al. 1987), deep brain stimulation (DBS) for movement disorders has become an efficient and widely used tool in the therapeutic armamentarium of various neurological conditions. Parkinson disease (PD), tremor, dystonia, epilepsy, and obsessive–compulsive disorder (OCD) are indications for treatment with deep brain stimulation. Table 1 summarizes approved indications for each system.

**Table 1:** CE approved indications for DBS systems

Manufacturer	Model	PD	Tremor	Dystonia	Epilepsy	OCD
B	Vercise	X	X	X		
	Vercise PC	X	X	X		
	Vercise Gevia	X	X	X		
M	Activa PC	X	X	X	X	X
	Activa RC	X	X	X		
	Activa SC	X	X	X		X
S	Libra	X	X	X		
	Libra XP	X	X	X		
	Brio	X	X	X		
	Infinity	X	X	X		

*PD* Parkinson disease, *OCD* obsessive–compulsive disorder (OCD), *B* Boston Scientific, *M* Medtronic, *S* St Jude

A typical DBS system consists of an electrode that is placed into the targeted cerebral structure, an implantable pulse generator (IPG), and an extension that connects the electrode to the IPG. The IPG itself consists of a case that houses a battery and electronic circuitry, which generates the electric signal going to the brain.

To achieve the desired therapeutic effect and to avoid undesired co-stimulation, the electric signal and the anatomical structures have to be aligned. This can be achieved by a well-shaped electric stimulation field, which can be adjusted using the DBS system.

In cases of perfectly implanted electrodes, the importance of the highly sophisticated technical features may be limited, but in cases where stimulation induces side effects, and those with little therapeutic window (defined as the difference between the amplitude of the electric signal at which side effects occur and the amplitude of the electric signal at which a significant therapeutic effect is observed), the availability of these features should be highly valued.

This review compares the technical features of DBS systems, deliberately limited to those approved for the European market. Information was primarily obtained from practical experience, promotional material, websites of the manufacturers, instructions for use, and competent support from representatives of the manufacturers. Altogether, DBS systems available from Boston Scientific (B), Medtronic (M) and St. Jude (S)^1^ are reviewed.

## Electrical sources

The electrical source is either a voltage source or a current source. Today the use of current sources is increasingly prevalent, but voltage sources represent the classical approach.

According to Ohm’s law, the current (*C*) is directly proportional to the voltage (*V*) and inversely proportional to the resistance (*R*). Depending on the electrical source, either the voltage or the current can be adjusted. The resistance depends on the electrical characteristics of the tissue, the electrode–tissue interface of the electrode itself, and in part on the stimulation. The resistance may vary over time, and it is not possible to anticipate the exact impedance behavior (Benabid et al. 1996; Hartmann et al. 2015).

With a voltage source, the desired voltage level can be adjusted. The resistance of the device and the anatomical structure results in a certain current value. This current value determines the therapeutic effect. The resistance can be subject to fluctuations due to subject- or device-related reasons. According to Ohm’s law, fluctuations in resistance result in fluctuations in the current. These current fluctuations can be avoided using a current source, as the current level can be adjusted directly (Bronstein et al. 2015).

All devices reviewed in this study can be operated with a current source. The devices marketed by B are equipped with multiple sources, with a dedicated current source for each contact on the electrode. The devices marketed by M also have the option to be operated using a voltage source. The technical features regarding current and voltage mode are summarized in Table 2.

**Table 2:** Stimulation option, electrical source and battery technology (rechargeable or non-rechargeable) of different DBS systems

Manufacturer	Model	Stimulation option	Electrical source	Rechargeable
B	Vercise	Unilateral/bilateral	Current	Yes
	Vercise PC	Unilateral/bilateral	Current	No
	Vercise Gevia	Unilateral/bilateral	Current	Yes
M	Activa PC	Unilateral/bilateral	Current/voltage	No
	Activa RC	Unilateral/bilateral	Current/voltage	Yes
	Activa SC	Unilateral	Current/voltage	No
S	Libra	Unilateral	Current	No
	Libra XP	Unilateral/bilateral	Current	No
	Brio	Unilateral/bilateral	Current	Yes
	Infinity	Unilateral/bilateral	Current	No

*B* Boston Scientific, *M* Medtronic, *S* St Jude

## Unilateral or bilateral systems

**Fig. 1:**
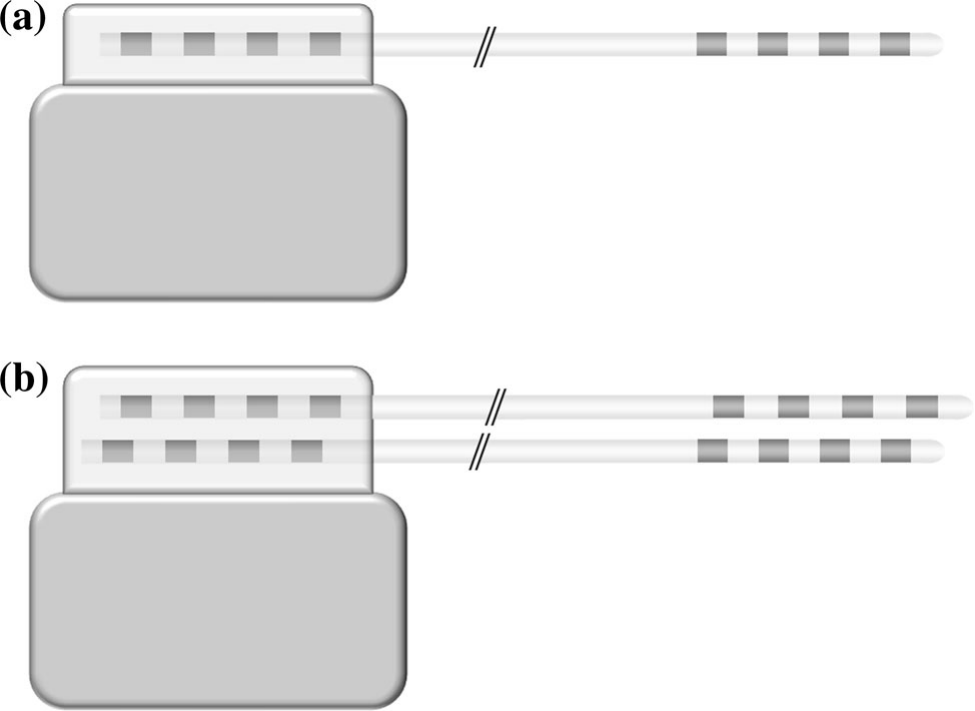
Schematic illustration of an IPG-lead combination used for **a** unilateral and **b** bilateral stimulation. The illustration shows electrodes with four contacts, whereas electrodes with eight contacts are also marketed

The systems can perform either unilateral or bilateral stimulation, depending on the number of connectors provided on the header of the IPG. To stimulate both hemispheres one can use two IPGs that offer the option of unilateral stimulation, or one IPG that offers the option of bilateral stimulation. A single IPG for bilateral stimulation was first implanted in 1998 (Vesper et al. 2002). Figure 1 shows a schematic illustration of an IPG and electrode combination for unilateral and bilateral stimulation. Table 2 summarizes this feature for each system provided by B, M and S.

## Stimulation field

A targeted and precise configuration of the electric stimulation field allows a localized neuronal response. The configuration, therefore, has a high impact on the efficacy of the therapy. Furthermore, the location of the electrode contacts in the brain target is essential for a potentially good therapy outcome.

The electric stimulation field depends on the distribution of the current in the targeted brain region, the pulse width, and the stimulation rate. The current distribution can be altered according to the electrode design, the polarity and proportion of current coming from each contact, and the amplitude. Thus, the technical features can be adapted to the requirements of the anatomical structure of the patient.

### Electrode design

The geometry of the electrode is a limiting factor for the desired current distribution, as the manufacturer fixes the number and design of contacts and the dimensions. The contact location in the targeted brain region is essential for optimizing the efficacy and minimizing side effects in DBS (Limousin et al. 1998; Herzog et al. 2004; Tripoliti et al. 2008). Newer contact designs allow directional stimulation with segmented contacts, which improves the possibility of targeted tissue activation and localized neuronal response (Steigerwald et al. 2016).

**Fig. 2:**
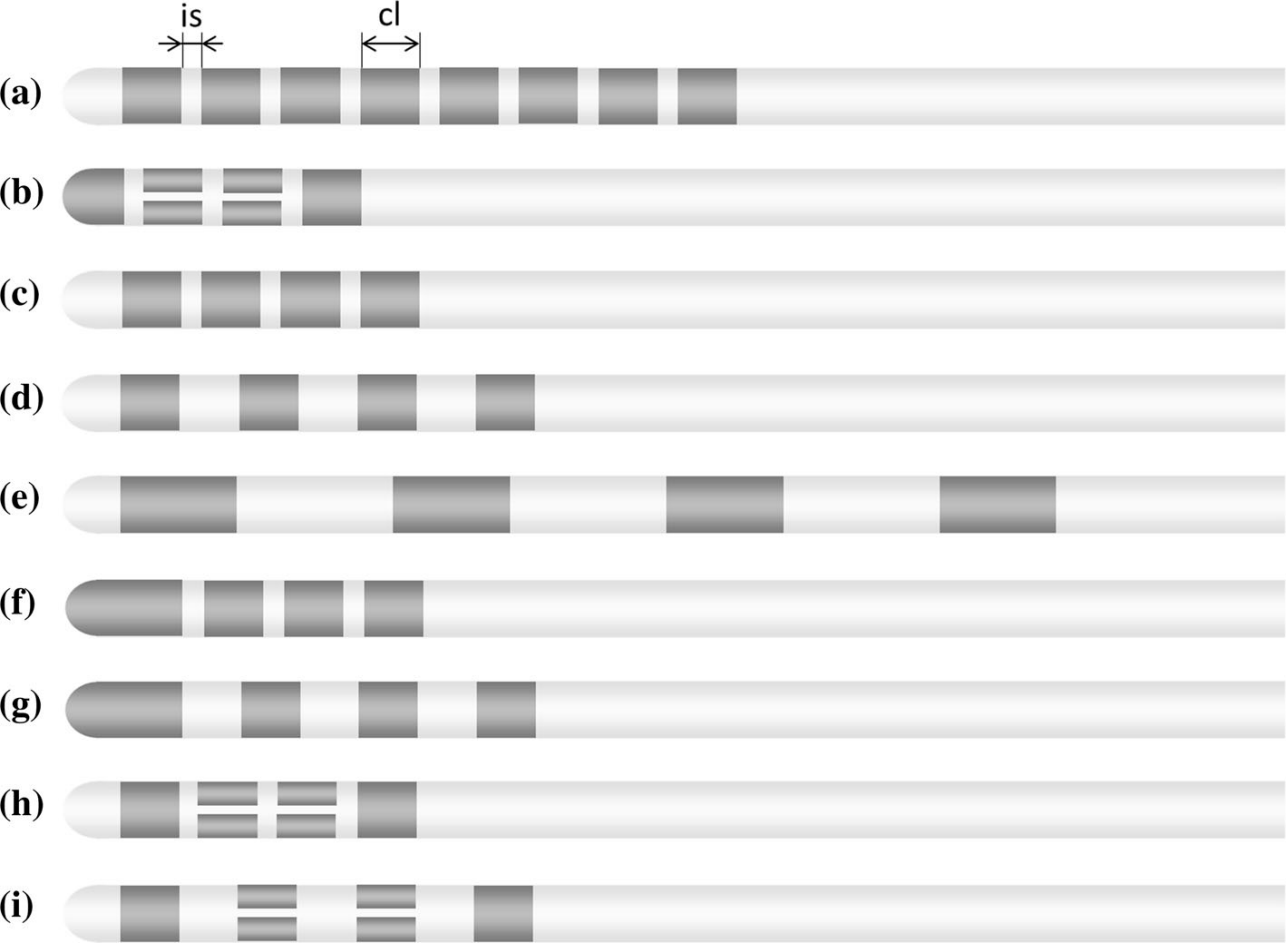
Illustration of the electrode contact configuration used in the target brain region. The number of contacts, contact length (cl), and intercontact spacing (is) are important design features that cannot be modified by the user

Electrodes are now marketed with four contacts (M and S) or eight contacts (B and S). Classical electrodes are equipped with ring contacts (B, M and S). Newer electrodes are also provided with segmented ring contacts (B and S). Electrodes with segmented rings contacts are equipped with ring contacts at most superior and the most inferior contact while the middle contacts are each split into three segments. The contact length (cl) is equal (1.5 mm) for all electrodes. The intercontact spacing (is) is either 0.5 mm (B, M, S) or 1.5 mm (M, S). Electrode designs are further specified in detail in Fig. 2 and Table 3.

**Table 3:** Dimensions of the electrode contact configuration

	Manufacturer	Lead model	is (mm)	cl (mm)	Number of contacts	Contact configuration
(a)	B	DB-2201-30AC/DB-2201-45BC	0.5	1.5	8	1-1-1-1-1-1-1-1
(b)	B	DB-2202-30/DB-2202-45	0.5	1.5	8	1-3-3-1
(c)	M	3389	0.5	1.5	4	1-1-1-1
(d)	M	3387	1.5	1.5	4	1-1-1-1
(e)	M	3391	4	3	4	1-1-1-1
(f)	S	6146/6147/6148/6149	0.5	1.5	4	1-1-1-1
(g)	S	6142/6143/6144/6145	1.5	1.5	4	1-1-1-1
(c)	S	6158/6160/6166/6168	0.5	1.5	4	1-1-1-1
(d)	S	6159/6161/6167/6169	1.5	1.5	4	1-1-1-1
(h)	S	6170/6172/6178/6180	0.5	1.5	8	1-3-3-1
(i)	S	6171/6173/6179/6181	1.5	1.5	8	1-3-3-1

All electrodes are used for the treatment of movement disorders except lead model 3391. Lead model 3391 is used for treatment of psychiatric disorders

*cl*, contact length, *is* intercontact spacing, *B* Boston Scientific, *M* Medtronic, *S* St Jude

### Polarity

**Fig. 3:**
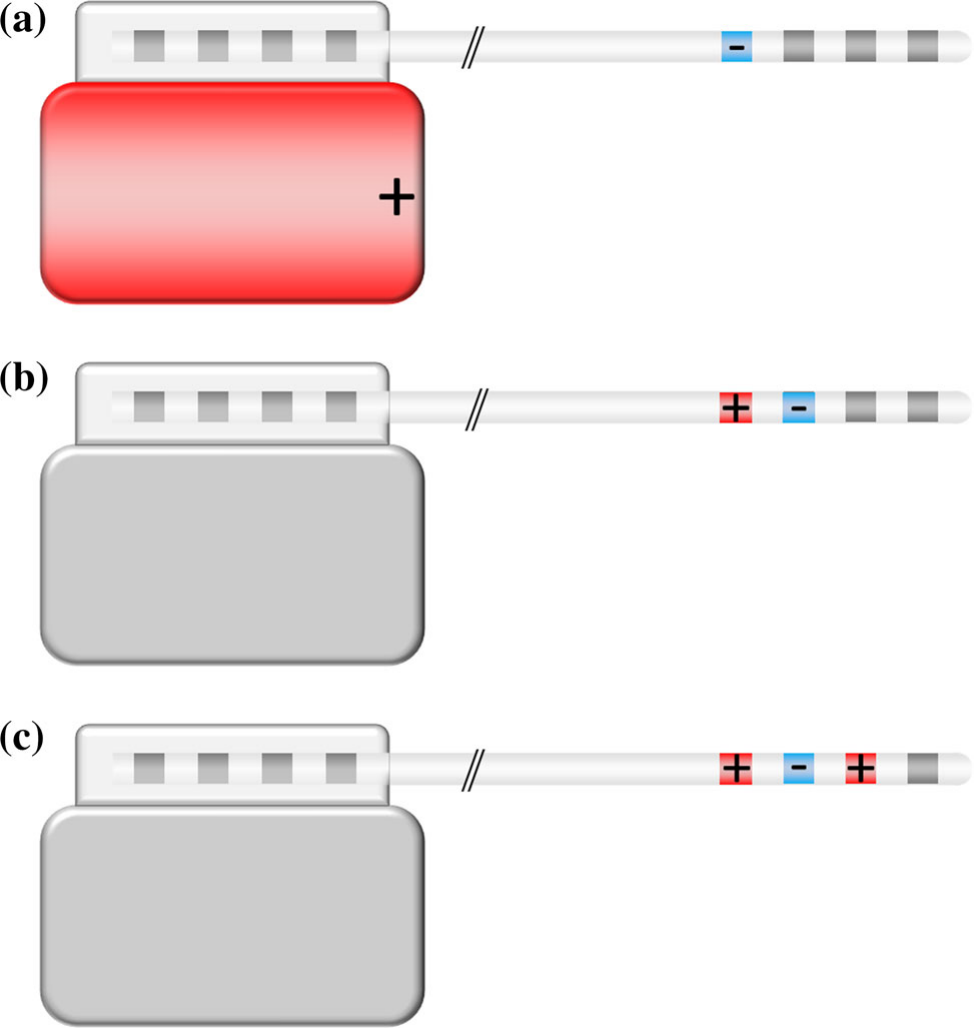
Schematic illustration of DBS systems operated in a **a** unipolar, **b** bipolar, and **c** multipolar configuration. In a unipolar configuration, the IPG acts as an anode (+) and one electrode contact as a cathode (−); in a bipolar configuration, both anode and cathode are located on one electrode contact each. A possible multipolar configuration is illustrated in **c**, with two anodes and one cathode

The polarity of the contacts is one factor that determines the distribution of the current in the targeted brain region. Both the contacts on the electrode and the case of the IPG can be programmed as cathode (−), anode (+), and off (neutral). In a monopolar configuration, one contact on the electrode is set to cathode and the case of the IPG acts as an anode. Bipolar stimulation is characterized by selecting one electrode contact as anode and another electrode contact as cathode. The case of the IPG is neutral in a bipolar stimulation setting. In a multipolar configuration, one or more electrode contact(s) are used as anode and one or more electrode contact(s) are used as cathode. The current flows between the cathode(s) and anode(s). The polarity configuration has an impact on the current flow, and therefore, on the volume of tissue activated (Butson and McIntyre 2008). Figure 3 shows a schematic representation of systems operated in unipolar, bipolar and multipolar configurations.

DBS systems commercialized by B and S provide unipolar, bipolar and multipolar configurations in current mode. The devices marketed by M provide unipolar, bipolar and multipolar configurations in voltage mode, but the polarity in the current mode is limited to unipolar and bipolar. Each system can assign the case of the IPG off (neutral) and with anodic polarity (+). Vercise PC and Vercise Gevia from B allow also programming the case as cathode (−). The characteristics of the reviewed systems are summarized in Table 4.

**Table 4:** Programmable characteristics of different DBS systems

Manufacturer	Model	Polarity	Amplitude	Pulse width (µs)	Rate (Hz)
B	Vercise	Uni-/bi-/multipolar	0–20 mA	10–450	2–255
	Vercise PC	Uni-/bi-/multipolar	0–20 mA	20–450	2–255
	Vercise Gevia	Uni-/bi-/multipolar	0–20 mA	20–450	2–255
M	Activa PC	Uni-/bipolar	0–25.5 mA	60–450	30–250
	Activa PC	Uni-/bi-/multipolar	0–10.5 V	60–450	2–250
	Activa RC	Uni-/bipolar	0–25.5 mA	60–450	30–250
	Activa RC	Uni-/bi-/multipolar	0–10.5 V	60–450	2–250
	Activa SC	Uni-/bipolar	0–25.5 mA	60–450	30–250
	Activa SC	Uni-/bi-/multipolar	0–10.5 V	60–450	2–250
S	Libra	Uni-/bi-/multipolar	0–12.75 mA	50–500	2–240
	Libra XP	Uni-/bi-/multipolar	0–12.75 mA	52–507	2–240
	Brio	Uni-/bi-/multipolar	0–12.75 mA	52–507	2–200
	Infinity	Uni-/bi-/multipolar	0–12.75 mA	20–500	2–240

*B* Boston Scientific, *M* Medtronic, *S* St Jude

### Electric signal

The electric signal stimulates the target area of the brain, and comprises the stimulation rate, the pulse width, and the stimulation amplitude. The stimulation rate specifies the number of pulses per second. The length of such a pulse is described by the pulse width, which can range between 10 and 507 μs. The stimulation amplitude defines the strength of the electric signal. When using a voltage source, the stimulation amplitude is given in voltage (V). The stimulation amplitude of current sources is given in milliamperes (mA). Efficacy and side effects are most affected by the stimulation amplitude and the stimulation rate (Moro et al. 2002; Kuncel et al. 2006). The electric signal may be further adapted to individual patient needs, by allocating different stimulation amplitudes to specific contacts (Timmermann et al. 2015). The pulse width may also affect the therapeutic effect. Data indicate that shorter pulse widths may be correlated with an increased therapeutic window (Reich et al. 2015).

**Fig. 4:**
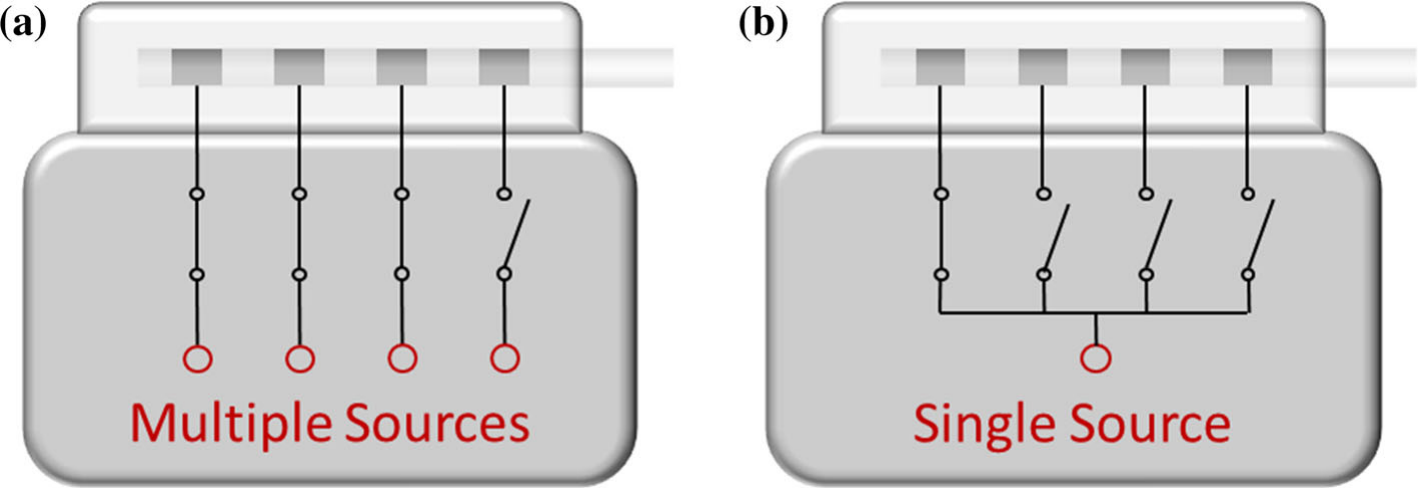
A DBS system may have a dedicated current source for each electrode contact (**a**) or a single current or voltage source that is shared by different electrode contacts (**b**)

Stimulation rate and pulse width can be set to a desired value, which is the same for all active contacts. The systems from B allow a specific amplitude level to be set for each contact on the electrode and for the case using multiple independent current sources (Fig. 4a). Systems from M and S are equipped with a single source (Fig. 4b). As a consequence all active contacts are provided with the same stimulation amplitude.

**Fig. 5:**
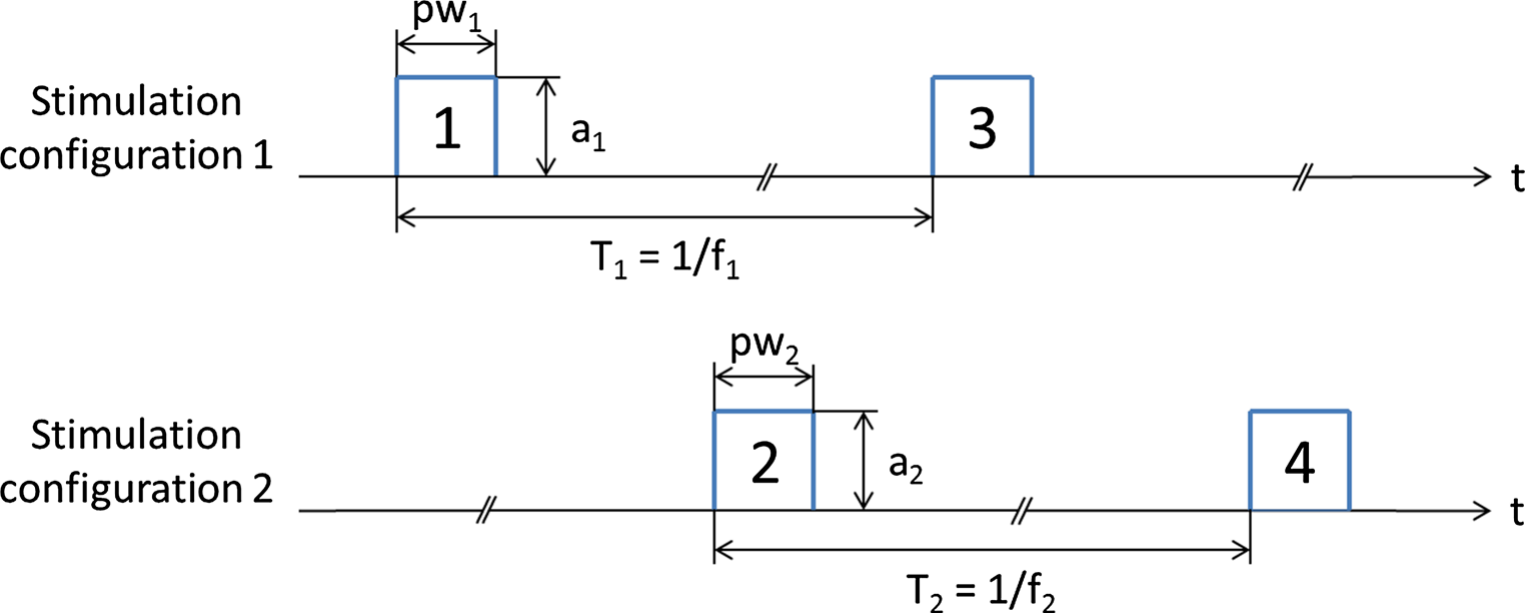
Illustration of an interleaving concept with two stimulation configurations. The stimulation configurations are executed in an interleaved mode. The electric signals are delivered sequentially. *a* amplitude, *pw* pulse width, *f* stimulation rate, *t* time

Stimulation pulses with different stimulation rate, pulse width, and stimulation amplitude can be achieved using different stimulation configurations (SC). The polarity setting and the electric signal form a SC. These configurations are executed in an interleaved mode. Figure 5 illustrates a configuration with two active SCs. First, electric signal 1 of configuration 1 is executed with an amplitude a1, and pulse width pw1. After that, the electric signal 2 of configuration 2 is delivered with an amplitude a2, and pulse width pw2. Then, electric signal 3 of configuration 1 is executed and so on. The stimulation rate of each configuration can be set according to the specifications of each system. Thereby, individualized current shaping can be achieved. This enables improved treatment of multiple symptoms while minimizing side effects, by variations of the stimulation site and electric signal (Wojtecki et al. 2011; Baumann et al. 2012; Kovacs et al. 2012; Weiss et al. 2013; Barbe et al. 2014; Ramirez-Zamora et al. 2015).

B allows four different SCs to be defined. The stimulation amplitude and the pulse width can be set separately for each SC. With the Vercise system, the stimulation rate can be different for each configuration if only two SCs are used. When using more than two SCs, only one stimulation rate can be used for all configurations. With Vercise PC and Vercise Gevia, different stimulation pulse widths, amplitudes, and stimulation rates can be chosen for all SCs. The contacts on the electrode can be configured as monopolar, bipolar and multipolar for each SC. With the Vercise system, one contact cannot be used in a monopolar mode in one SC and in a multipolar mode in another SC. Vercise PC and Vercise Gevia do not have this limitation.

M also provide systems that allow programming of four SCs. Two SCs can be defined for each hemisphere. The stimulation amplitude, the pulse width, and the polarity of the contacts can be adjusted for each SC. The stimulation rate is the same for all SCs. M calls the option of running different SCs on a DBS system interleaving stimulation.

## Battery

DBS systems are designed as either rechargeable or non-rechargeable. Non-rechargeable systems store energy for the complete lifetime of the battery. The longevity of the systems depends on parameters used for the electric signal, such as stimulation amplitude, stimulation rate, pulse width, the number of active contacts, and the battery capacity. Furthermore, the energy consumption of the device itself, such as the electronic circuit or impedances of the electrode, affects battery longevity. Rechargeable systems have to be recharged on a regular basis. Rechargeable and non-rechargeable systems currently marketed are summarized in Table 2.

## Programmers

The stimulation field can be set and adjusted using a programmer (Volkmann et al. 2006). The desired parameters for the stimulation field are transmitted from the programmer to the IPG by radio frequency coupling. B, M and S provide their own programmers developed for physicians and patients. The Infinity system provided by S uses Apple digital devices for its programmers, which have a Bluetooth link to communicate with the IPG.

## Interconnectivity

The rising diversity of DBS systems also focuses attention on the interconnectivity of different DBS systems. While geometrical aspects of the connectors are very similar, the design is specific and does not allow a simple connection.

DBS systems consist of an electrode, an IPG and an extension that connects the electrode to the IPG. Each manufacturer provides these components for its own DBS systems. The combination of components from different manufacturers can be achieved using adaptors. These adaptors connect the extension to the IPG and thereby allow the use of different electrode-IPG-combinations. Electrodes from M can potentially be connected to any IPG from B, M and S. However, no adaptors are provided to connect the electrodes from B and S to IPGs from M (Table 5). Additionally, adaptors are currently also offered for electrodes used with the classical IPGs provided by M (Itrel, Soletra, Kinetra). These IPGs are not on the market anymore, but patients may have them implanted. In case of a replacement, IPGs from different manufacturers can be connected with an adaptor to the implanted electrodes.

**Table 5:** Possible interconnections between components from different manufacturers

Manufacturer	IPG-model	B: DB-2201-30AC/DB-2201-45BC	B: DB-2202-30/DB-2202-45	M: 3387	M: 3389	M: 3391	S: 6146/6147/6148/6149	S: 6142/6143/6144/6145	S: 6158/6160/6166/6168	S: 6159/6161/6167/6169	S: 6170/6172/6178/6180	S: 6171/6173/6179/6181
B	Vercise	X		A	A							
	Vercise PC		X	A	A							
	Vercise Gevia	X	X	A	A							
M	Activa PC			X	X	X						
	Activa RC			X	X							
	Activa SC			X	X	X						
S	Libra			A	A		X	X	X	X	X	X
	Libra XP			A	A		X	X	X	X	X	X
	Brio			A	A		X	X	X	X	X	X
	Infinity			A^a^	A^a^		X	X	X	X	X	X

The top row refers to the electrode models. (X) indicates devices that may be connected directly, without any further material. (A) indicates possible connections with adaptors

*B* Boston Scientific, *M* Medtronic, *S* St Jude

^a^Medtronic extension model 7483 can be connected via an adaptor, and extension model 37086 can be connected directly

## Discussion

This review focuses on technical features that are primarily relevant for electrical stimulation. The systems may differ with regard to electrical source, possible stimulation regions, and the stimulation field. When DBS stimulation was first introduced, voltage-only sources were used. In recent years, current sources have been used more often. All DBS systems currently on the market provide current sources. The number of electrode contacts has increased from four contacts to up to eight contacts. In the latest developments in electrode design, one single contact is segmented into three contacts. This allows the current distribution to be adjusted and directed more specifically. The possible settings for the stimulation rate, the pulse width, and the stimulation amplitude have not changed significantly over time, and most of the DBS systems may be operated in a uni-, bi- and multipolar configuration. Some manufacturers also provide the option to adjust specific settings with the concept of interleaving stimulation, which can reflect specific patient requirements. Nevertheless, the setting options are limited with regard to the possible stimulation rates and polarity configurations of the electrode contacts. In the past, only non-rechargeable systems were marketed; today, all manufacturers provide rechargeable and non-rechargeable systems. The diversity of DBS systems also raises the question on interconnectivity between components of different manufacturers. The combination of technical features of an impulse generator from one manufacturer and an electrode from another manufacturer may be a desirable requirement in clinical practice. Consequently, a uniform standard would be required to simplify efforts for interconnecting components from different manufacturers.

The DBS systems appear quite similar, but when it comes to technical features and their combination systems differ significantly from each other. To achieve the best therapeutic effect, technical features, its combination possibilities, and the limitations of the systems have to be considered.

## Footnotes

1 Abbott acquired St. Jude early 2017.

## References

[CR1] Barbe MT, Dembek TA, Becker J, Raethjen J, Hartinger M, Meister IG, Runge M, Maarouf M, Fink GR, Timmermann L (2014). Individualized current-shaping reduces DBS-induced dysarthria in patients with essential tremor. Neurology.

[CR2] Baumann CR, Imbach LL, Baumann-Vogel H, Uhl M, Sarnthein J, Surucu O (2012). Interleaving deep brain stimulation for a patient with both Parkinson’s disease and essential tremor. Mov Disord.

[CR3] Benabid AL, Pollak P, Louveau A, Henry S, de Rougemont J (1987). Combined (thalamotomy and stimulation) stereotactic surgery of the VIM thalamic nucleus for bilateral Parkinson disease. Appl Neurophysiol.

[CR4] Benabid AL, Pollak P, Gao D, Hoffmann D, Limousin P, Gay E, Payen I, Benazzouz A (1996). Chronic electrical stimulation of the ventralis intermedius nucleus of the thalamus as a treatment of movement disorders. J Neurosurg.

[CR5] Bronstein JM, Tagliati M, McIntyre C, Chen R, Cheung T, Hargreaves EL, Israel Z, Moffitt M, Montgomery EB, Stypulkowski P, Shils J, Denison T, Vitek J, Volkman J, Wertheimer J, Okun MS (2015). The rationale driving the evolution of deep brain stimulation to constant-current devices. Neuromodulation.

[CR6] Butson CR, McIntyre CC (2008). Current steering to control the volume of tissue activated during deep brain stimulation. Brain Stimul.

[CR7] Hartmann CJ, Wojtecki L, Vesper J, Volkmann J, Groiss SJ, Schnitzler A, Sudmeyer M (2015). Long-term evaluation of impedance levels and clinical development in subthalamic deep brain stimulation for Parkinson’s disease. Parkinsonism Relat Disord.

[CR8] Herzog J, Fietzek U, Hamel W, Morsnowski A, Steigerwald F, Schrader B, Weinert D, Pfister G, Muller D, Mehdorn HM, Deuschl G, Volkmann J (2004). Most effective stimulation site in subthalamic deep brain stimulation for Parkinson’s disease. Mov Disord.

[CR9] Kovacs N, Janszky J, Nagy F, Balas I (2012). Changing to interleaving stimulation might improve dystonia in cases not responding to pallidal stimulation. Mov Disord.

[CR10] Kuncel AM, Cooper SE, Wolgamuth BR, Clyde MA, Snyder SA, Montgomery EB, Rezai AR, Grill WM (2006). Clinical response to varying the stimulus parameters in deep brain stimulation for essential tremor. Mov Disord.

[CR11] Limousin P, Krack P, Pollak P, Benazzouz A, Ardouin C, Hoffmann D, Benabid AL (1998). Electrical stimulation of the subthalamic nucleus in advanced Parkinson’s disease. N Engl J Med.

[CR12] Moro E, Esselink RJ, Xie J, Hommel M, Benabid AL, Pollak P (2002). The impact on Parkinson’s disease of electrical parameter settings in STN stimulation. Neurology.

[CR13] Ramirez-Zamora A, Kahn M, Campbell J, Delacruz P, Pilitsis JG (2015). Interleaved programming of subthalamic deep brain stimulation to avoid adverse effects and preserve motor benefit in Parkinson’s disease. J Neurol.

[CR14] Reich MM, Steigerwald F, Sawalhe AD, Reese R, Gunalan K, Johannes S, Nickl R, Matthies C, McIntyre CC, Volkmann J (2015). Short pulse width widens the therapeutic window of subthalamic neurostimulation. Ann Clin Transl Neurol.

[CR15] Steigerwald F, Muller L, Johannes S, Matthies C, Volkmann J (2016). Directional deep brain stimulation of the subthalamic nucleus: a pilot study using a novel neurostimulation device. Mov Disord.

[CR16] Timmermann L, Jain R, Chen L, Maarouf M, Barbe MT, Allert N, Brucke T, Kaiser I, Beirer S, Sejio F, Suarez E, Lozano B, Haegelen C, Verin M, Porta M, Servello D, Gill S, Whone A, van Dyck N, Alesch F (2015). Multiple-source current steering in subthalamic nucleus deep brain stimulation for Parkinson’s disease (the VANTAGE study): a non-randomised, prospective, multicentre, open-label study. Lancet Neurol.

[CR17] Tripoliti E, Zrinzo L, Martinez-Torres I, Tisch S, Frost E, Borrell E, Hariz MI, Limousin P (2008). Effects of contact location and voltage amplitude on speech and movement in bilateral subthalamic nucleus deep brain stimulation. Mov Disord.

[CR18] Vesper J, Chabardes S, Fraix V, Sunde N, Ostergaard K, Kinetra Study G (2002). Dual channel deep brain stimulation system (Kinetra) for Parkinson’s disease and essential tremor: a prospective multicentre open label clinical study. J Neurol Neurosurg Psychiatry.

[CR19] Volkmann J, Moro E, Pahwa R (2006). Basic algorithms for the programming of deep brain stimulation in Parkinson’s disease. Mov Disord.

[CR20] Weiss D, Walach M, Meisner C, Fritz M, Scholten M, Breit S, Plewnia C, Bender B, Gharabaghi A, Wachter T, Kruger R (2013). Nigral stimulation for resistant axial motor impairment in Parkinson’s disease? A randomized controlled trial. Brain.

[CR21] Wojtecki L, Vesper J, Schnitzler A (2011). Interleaving programming of subthalamic deep brain stimulation to reduce side effects with good motor outcome in a patient with Parkinson’s disease. Parkinsonism Relat Disord.

